# Design, implementation and evaluation of a training programme for school teachers in the use of malaria rapid diagnostic tests as part of a basic first aid kit in southern Malawi

**DOI:** 10.1186/s12889-015-2228-x

**Published:** 2015-09-17

**Authors:** Stefan Witek-McManus, Don P. Mathanga, Allison Verney, Austin Mtali, Doreen Ali, John Sande, Reuben Mwenda, Saidi Ndau, Charles Mazinga, Emmanuel Phondiwa, Tiyese Chimuna, David Melody, Natalie Roschnik, Simon J. Brooker, Katherine E. Halliday

**Affiliations:** Save the Children International, Zomba, Malawi; Faculty of Infectious and Tropical Diseases, London School of Hygiene & Tropical Medicine, London, UK; Malaria Alert Centre, College of Medicine, University of Malawi, Blantyre, Malawi; National Malaria Control Programme, Ministry of Health, Lilongwe, Malawi; Health Technical Support Services-Diagnostics, Ministry of Health, Lilongwe, Malawi; Zomba District Health Office, Ministry of Health, Zomba, Malawi; Department of School Health, Nutrition, HIV & AIDS, Ministry of Education, Science & Technology, Lilongwe, Malawi; Zomba District Education Office, Ministry of Education, Science & Technology, Zomba, Malawi; Save the Children USA, Washington, DC USA

## Abstract

**Background:**

With increasing levels of enrolment, primary schools present a pragmatic opportunity to improve the access of school children to timely diagnosis and treatment of malaria, increasingly recognised as a major health problem within this age group. The expanded use of malaria rapid diagnostic tests (RDTs) and artemisinin combination therapy (ACT) by community health workers (CHWs) has raised the prospect of whether teachers can provide similar services for school children. We describe and evaluate the training of primary school teachers to use a first aid kit containing malaria RDTs and ACT for the diagnosis and treament of uncomplicated malaria in school children in southern Malawi.

**Methods:**

We outline the development of the intervention as: (1) conception and design, (2) pilot training, (3) final training, and (4) 7-month follow up. The training materials were piloted at a four-day workshop in July 2013 following their design at national stakeholders meetings. The evaluation of the pilot training and materials were assessed in relation to increased knowledge and skill sets using checklist evaluations and questionnaires, the results of which informed the design of a final seven-day training programme held in December 2013. A follow up of trained teachers was carried out in July 2014 following 7 months of routine implementation. A total of 15 teachers were evaluated at four stages: pilot training, two weeks following pilot, final training and seven months following final training.

**Results:**

A total of 15 and 92 teachers were trained at the pilot and final training respectively. An average of 93 % of the total steps required to use RDTs were completed correctly at the final training, declining to 87 % after 7 months. All teachers were observed correctly undertaking safe blood collection and handling, accurate RDT interpretation, and correct dispensing of ACT. The most commonly observed errors were a failure to wait 20 minutes before reading the test result, and adding an incorrect volume of buffer to the test cassette.

**Conclusion:**

Following training, teachers are able to competently use RDTs and ACTs test and treat children at school for uncomplicated malaria safely and accurately. Teachers demonstrate a comparable level of RDT use relative to non-health professional users of RDTs, and sustain this competency over a period of seven months during routine implementation.

## Background

In the treatment of malaria, the World Health Organization (WHO) recommends parasitological confirmation prior to administration of anti-malarial drugs [1]. Malaria rapid diagnostic tests (RDTs) provide a reliable and practical means to provide diagnosis and are now routinely used by health workers [2]. There is increasing effort in incorporating RDTs across a wider spectrum of providers [3], embracing community health workers as part of community case management of malaria [4–6], and in both the formal and informal private sector [7–9]. As consequence, there has been recent interest in training community health workers (CHW) to use RDTs [10] and in introducing RDTs at private drug shops [11]. Previous studies have shown that CHWs can accurately use RDTs after receiving training and a set of pictorial instructions [6, 12–14] and that this competence is retained over 12 months [15]. However, some reservations remain regarding the use of RDTs by non-health professionals due to concerns about blood safety, ability to interpret the test results correctly, and inappropriate use of ACTs [16, 17].

Malaria among school-aged children is increasingly being recognised as an important public health challenge [18–21], yet geographical and financial barriers that prevent school-aged children from obtaining prompt access to diagnosis and effective treatment remain [22–24]. Notably, recent evidence from Malawi has reported school-aged children to be at higher risk of *Plasmodium* infection than younger children, while also less likely to be brought for any type of malaria treatment [25]. Malawi has previously deployed school-based first aid kits (known as "Pupil Treatment Kits" or "PTKs") to provide immediate treatment for common health problems, including presumptive diagnosis and treatment of malaria using SP. One aim of the PTK was to reduce absenteeism attributable to malaria by providing prompt treatment at school, demonstrated by a reduction in overall and malaria-specific mortality [26] and decreased absenteeism and grade repetition [27]. Previous first-line treatments, such as chloroquine or sulphadoxine-pyramethamine, did not require parasitological diagnosis and highlighted the feasibility and effectiveness of training of teachers to provide presumptive treatment [28, 29]. However, the introduction of artemisinin-based combination therapy (ACTs) in the country and the associated need for parasitological diagnosis led to the withdrawal of SP from PTKs shortly after beginning national rollout in 2008. The expanded use of RDTs by non-health professionals provides the opportunity to revisit the role that teachers can play in the diagnosis and treatment of uncomplicated malaria.

This paper documents the process of training primary school teachers in southern Malawi to use a first aid kit (hereby referred to as a “Learner Treatment Kit” or “LTK”) containing malaria RDTs and ACT for the diagnosis and treatment of uncomplicated malaria in primary school children. The LTK is currently the subject of a broader cluster randomised trial across 58 primary schools within TA Chikowi investigating the effectiveness, cost-effectiveness and feasibility of such an intervention (ClinicalTrials.gov: NCT02213211). As part of this trial, an evaluation of the LTK training was undertaken to assess whether trained teachers could accurately and safely use RDTs and provide appropriate treatment with ACT, and whether this competence was retained up to 7 months after training during routine implementation.

## Methods

### Study context

A school-based programme of malaria diagnosis and treatment is currently being implemented by the Ministry of Health (MoH), Ministry of Education, Science & Technology (MoEST) and Save the Children. Between 2000 and 2007, a School Health and Nutrition (SHN) programme was implemented in primary schools in Mangochi district, where malaria was presumptively treated by teachers for malaria using sulphadoxine pyrimethamine (SP). Approximately 11,000 children across 100 primary schools were treated for uncomplicated malaria over the course of each year until the withdrawal of SP as first line treatment of uncomplicated malaria in 2008.

Since 2012, Save the Children has been collaborating with the district health and education authorities encompassing the Traditional Authority (TA) Chikowi in Zomba District in southern Malawi, including initiatives to investigate the burden of malaria in school children and potential approaches for its control. TA Chikowi is a rural, malaria endemic area [30]. Net primary school enrolment was estimated to be 87 % as of 2007 [31]. School surveys conducted in 50 schools in 2011 revealed that 60 % of children were infected with *Plasmodium falciparum* malaria parasites and 32 % were anaemic [Mathanga et al. 2015, in press]. This survey also found that 38 % of school children report usually sleeping under any type of mosquito net, although this may have increased following a subsequent national long lasting insecticidal net (LLIN) distribution campaign targeting at all age groups conducted by the NMCP in 2012. Awareness of this burden ultimately led the NMCP of Malawi, in discussion with the MoEST and Save the Children to consider reintroducing the PTK (now renamed as LTK). Since 2008, WHO guidelines recommend parasitological confirmation of all suspected cases of malaria prior to anti-malarial treatment [1] and as such, LTKs would now be required to incorporate both RDTs and ACTs. This programme complements the current piloting of RDTs for community case management (CCM) of malaria in children under 5 years by health surveillance assistants in Malawi.

### Learner Treatment Kit

The Learner Treatment Kit is a simple first aid kit, intended to be available to all primary school children (approximate age range 4–18 years) during school hours for the management of basic health problems, including uncomplicated malaria. At each school, between two and four trained teachers (called LTK dispensers) received seven days training in the use of the LTK, followed by a three-day mentorship period at a local health centre, as well as on-going support from the implementing partner. Alongside RDTs and ACTs, the LTK includes a limited selection of basic supplies including oral rehydration salts, tetracycline ointment, paracetamol and wound and burn dressings, as well as malaria-testing specific materials (e.g. sharps bin) and general items (e.g. gloves, weighing scales, waste bin). The LTK box is a double locked wooden box, kept in a locked room in the school to which only trained LTK dispensers have keys to access the contents. Non-sharps biowaste is burned on school grounds and sharps biowaste is disposed of in specially constructed locked pit latrines used solely for this purpose.

The LTK programme aims to encourage prompt treatment seeking by school children for any health problem at a place that is convenient, safe and acceptable. By improving the management of common health problems experienced by school children, such as malaria, the LTK is envisaged to reduce the number of days lost due to absenteeism from school. All services of the LTK are provided free of charge. In the event of any complicated or urgent health complaint, school children are immediately referred to a local health centre. LTK dispensers do not directly accompany the child to the health centre, but will call for a caregiver and explain why they have referred the child.

### Development of training materials and programme

A likely difference between training health workers to use RDTs and training teachers is that the former have familiarity with general healthcare principles, including biosafety. It was therefore considered essential to develop a comprehensive and skill-focused training that would enable teachers to use RDTs and ACTs safely and accurately. The design of the training and production of associated materials was developed through a series of meetings held between June 2012 to July 2013 by a technical working group comprised of national, regional and district level parties from MoH and MoEST, with support from Save the Children, College of Medicine - University of Malawi and London School of Hygiene & Tropical Medicine. The basis of the LTK manual and training materials was current MoEST national school health curriculum, which includes a brief training for teachers in general issues relating to school child health and nutrition and is intended to complement the availability of basic school first aid kit. With the inclusion of RDT and ACTs, community case management documents used by MoH were incorporated. These materials were then refined in specific aspects of the LTK by incorporating relevant aspects of archive documents (e.g., manual from the implementation of the first generation of PTKs), contemporary training materials developed by the ACT Consortium for the training shop-keepers and community health workers in the use of RDTs in Uganda [48], and illustrated job-aids developed for use by community health workers developed by WHO [32]. The final LTK training materials are outlined in Table 1 and the full material can be accessed from [http://www.thiswormyworld.org/learner-treatment-kit-project].

**Table 1:** Outputs of technical working group

Document	Description
Teachers’ manual	Illustrated manual containing all information pertaining to the LTK, including guide to basic health problems, use and management of supplies, and record-keeping.
Illustrated job-aids	Five job aids designed to support LTK dispensers in (1) criteria for using an RDT (2) danger signs (symptoms) requiring referral to health centres (3) how to conduct an RDT (4) interpreting RDT results (5) treatment regimens for AL, paracetamol and ORS.
Treatment register	Based on design of village health clinic registers as currently used in Malawi, completed for every learner consulted by the LTK dispenser.
Monthly reporting form	As required by district health office for monthly reporting; outlining summaries of health problems seen, treatments given and consumption of supplies.
Stock order form	Completed by LTK dispensers when requesting supplies from a dispensing health centre.
Referral form	For the referral of all learners displaying complicated or emergency ‘signs’ that are not managed using the LTK.
Treatment information forms	To support verbal instructions given to learner; information sheet translated into main local language to explain symptoms observed and management and/or advice given.

### Training of teachers

A pilot training was conducted in July 2013 with 15 teachers from five purposively selected primary schools lying outside of TA Chikowi (the ultimate implementation area). The head teacher of each school was asked to identify one male and one female teacher to participate in the training. Participants were invited to attend a residential 4.5 day training (including a half-day evaluation) on the use of the LTK. The training was delivered by four facilitators from the District Health Office (DHO), all of whom had previously participated in training health workers in the management of malaria based on the use of RDTs. During the pilot training, 14 hours (h) were spent on the diagnosis and management of malaria across the four days, with introduction to malaria and its control (1.5 h), steps in diagnosis and treatment of malaria (4 h), practical demonstration of RDT use (3 h), RDT practice by the teachers (3.5 h) and completing the treatment documentation (2 h). An evaluation of the pilot training was used to inform the final design of the training programme.

On the basis of the results of the pilot training evaluation, four key modifications were made to the structure of the training programme: (i) increased training hours in practice of conducting RDTs, (ii) a day-long field visit (under facilitator supervision) for teachers to experience conducting malaria testing and treatment in the school environment, (iii) increased training hours in treatment of malaria, including documentation, and (iv) additional emphasis and practice in recognising danger signs and the process of referral. This increased the total length of the training workshop from four and a half to seven days. Schools were advised to select teachers below the age of fifty years for the training, with the exception of the head teacher who regardless of age was trained to act as a supervisor. On completion of training, teachers were required to complete a three day mentorship period at their local health facility to observe RDTs being conducted on patients who required testing for malaria.

The final training consisted of the following components: (i) introduction to the learner treatment kit (3 h), (ii) introduction to malaria and its control (3 h) (iii) steps involved in the diagnosis and treatment of malaria (3 h), (iv) practical demonstration on conducting an RDT (3 h), (v) practice in the use of an RDT (12 h, including a one-day field visit to a study primary school), (vi) management of (non-malaria) minor illnesses and injuries (6 h) and (vii) treatment decisions and record keeping (4 h).

The final programme training was conducted in two phases during November and December 2013. In 28 randomly selected primary schools in TA Chikowi, 2 or 3 teachers (depending on school enrolment) plus the head teacher were invited to attend training to be a LTK dispenser, using criteria established by the District Education Office for the selection of teachers to carry out SHN activities. In total, 92 teachers attended a 7 day training programme (Table 2), delivered by thirteen facilitators from national, zonal and district health offices; including three who had attended the pilot training. Teachers were invited to attend one of two identical training sessions with no more than 50 teachers per session, shared equally across two separate classrooms. Facilitator’s time was generally split between the two classrooms, moving between depending on teaching topic.

**Table 2:** Demographic and professional characteristics of pilot training and final training participants

	%, median or mean (range)			
Characteristic:	Pilot training participants and evaluation sample (*n* = 15)	Final training		
		All participants (*n* = 92)	End of training evaluation sample (*n* = 15)	7 month follow-up sample (*n* = 15)
Male (%)	60	68	67	80
Headteacher (%)	33	32	33	13
Median age (years):	37 (21–58)	31 (21–58)	30 (24–49)	30 (24–45)
Median length of employment(years):	4 (0.5–33)	4 (0.75–35)	4 (1.5–24)	1.5 (1.5–24)
> 5 years’ experience (%):	47	38	53	27
Classes currently taught (%):				
None	13	0	0	0
Standard 1–4	13	40	43	40
Standard 5–8	80	64	64	67
Subjects taught (%):				
Life Skills	66	55	38	47
English	66	57	38	47
Science	40	30	38	27
Mathematics	40	69	64	73

### Evaluation of training

After both the pilot training and the main programme training, 15 teachers were assessed in their ability to conduct RDT procedures safely and accurately, and to interpret test results accurately. At the pilot training all teachers participated in the evaluation, whereas a sample of 15 teachers from all final training participants were randomly selected for evaluation. Teachers’ ability to conduct test procedures safely and accurately was assessed using a 26 item checklist of steps included in test use, adapted from previous evaluations of RDT use [10, 12, 15, 33–36]. Each individual step was classified as being completed correctly (i.e. as described), incorrectly or not at all. For the purpose of analysis, the results of each checklist were further grouped into steps considered to be necessary to conduct an RDT safely (steps 3, 8, 10, 12 and 16) and accurately (steps 14, 15, 17, 18, 20a/20b and 21) as similarly described by Counihan *et al.* [15] (Table 3). Teacher’s ability to interpret RDTs accurately was assessed using a printed sheet of five RDTs of differing results [37]. The ability to give appropriate treatment was assessed using a short case-scenario where teachers were required to select the appropriate treatment dose and complete the learner treatment register and parent information sheet based on a positive RDT result, weight and age of the child, and reported minor symptoms. Finally, a self-administered questionnaire recorded personal and professional characteristics of the teachers.

**Table 3:** Analysis of RDT use and treatment scenario steps (i) following completion of pilot training and (ii) 2 weeks after pilot training, by individual step

			(% completing step correctly) (*n* = 15)		
#		Description of step	End of training	2 weeks post training	Common error(s)
1		Work surface disinfected with alcohol	100	93	
2		Assembles all required equipment	47	87	Lack of preparation of items before conducting RDT
3	S	Put on new pair of gloves	100	100	
4		Check expiry date of RDT packet	80	73	Skipped
5		Check desiccant sachet is dry	40	66	Skipped
6		Write client name on cassette	100	100	
7		Place cassette on level surface	100	100	
8	S	Clean finger with alcohol swab	93	100	
9		Allow finger to dry before pricking	100	100	
10	S	Use sterile lancet to prick finger	93	93	Set down sterile lancet onto work surface after removing cap
11		Puncture side of ball of the 3rd or 4th finger	93	100	
12	S	Dispose of lancet immediately after pricking finger	87	100	Used lancet, then set down onto work surface
13		Wipes away initial blood from finger	93	93	
14	A	Uses blood collection device correctly	73	100	Attempted to ‘scoop’ blood, inadequate volume of blood
15	A	Correctly transfer blood to cassette using tool	93	100	
16	S	Dispose of blood collection tool immediately	100	100	
17	A	Dispenses correct volume of buffer	93	93	
18	A	Start the timer immediately after adding buffer	60	66	Started timer before adding buffer or after clearing workspace
19		Dispose of non-sharps safely	100	100	
20	A	State the correct time that the RDT can be read	80	87	Stated “15 to 20” or “20 minutes”
21	A	Reads test results correctly (RDT test sheet)	53	87	Misidentified faint positive as negative or invalid.
22	T	Completes correct sections of treatment register	87		Not completed
23	T	Selects appropriate AL dose based on weight	60		Ticks “LA x 1 tab” on treatment register
24	T	Selects appropriate paracetamol dose	60		Not completed; prescribes incorrect dose
25	T	Selects correct Parent Information sheet for AL dose	13		Uses LA x 1 Parent Information Sheet
26	T	States correct time for 2nd dose of AL to be taken	40		Not completed; instructed 2nd dose to be taken after 6 hours

*S* steps relating to safety of conducting test, *A* steps relating to accuracy of conducting test, *T* steps relating to treatment of uncomplicated malaria

Two weeks after the pilot training, teachers were re-evaluated using the same checklist to assess retention of skills in using RDTs. Seven months following the final programme trained teachers were followed up during routine visits to schools to assess whether safety and accuracy remained adequate over time. This evaluation was conducted in-situ, with teachers observed at their school testing a child who had sought assistance on that day.

Teachers at the pilot training were further required to complete a script concordance test (SCT) consisting of a short clinical vignette, following which the teacher was presented with at least two independent hypotheses, each with an associated ‘new’ piece of information that they were required to interpret [38]. The teacher was required to judge the effect of this new information using a five-point Likert scale against the original diagnostic hypothesis. The similarity, or concordance, of this response was then measured against that of a panel of experts (in this case, the training facilitators) whose responses were aggregated [39]. For the purpose of evaluation, this SCT was restricted to ten diagnostic or treatment scenarios with two or three potential hypotheses. A previously proposed method of assessment was used to interpret scores, whereby the scores of the teachers were interpreted based on the number of standard deviations from the mean score given by the panel of experts [40]. Results were considered acceptable if they were within four standard deviations of the mean of the expert panel, based on a previously described threshold [41].

Finally, perceptions and opinions of the pilot training workshop agenda, presentations and materials were assessed through focus group discussions and self-administered questionnaires completed by the teachers and semi-structured interviews also took place with three of the facilitators. Topics of discussion were divided into two areas: the training programme as a whole, and training specific to use of RDTs.

### Data analysis

Results of evaluation checklists and script-concordance tests were collated, inputted into Excel 2013 (Microsoft, Redmond, WA) and analysed using Stata 13 (Statacorp, College Station, TX). The percentage of steps performed correctly after the final training and seven months later, and differences in the proportions of teachers completing the RDT safely or accurately were analysed using Fishers’s exact test. Due to *a priori* concerns over the age of teachers trained to become LTK dispensers, further analysis was conducted on the pilot training results comparing the results between two age groups, divided along the median pilot training participant age (37 years). The results of the SCT conducted in the pilot training was analysed using the Mann-Witney *U* test. The internal consistency of the SCT was acceptable (Cronbach’s α = 0.76). Differences in the demographic characteristics of each evaluation sample were assessed by Kruskal-Wallis test.

Focus group discussions and individual interviews conducted were transcribed and managed using Nvivo 10 (QSR International Pty Ltd, Australia). Coding took place collectively across all qualitative data sources to identify key themes, both within and across different data collection tools. Data were thematically analysed to describe experiences of the pilot training programme from both participant and facilitators perspectives, and were interpreted in the context of the results of the other evaluation tools.

### Ethical approval

The study was approved by the National Health Sciences Research Committee (NHSRC) Malawi (IRB No. 1057) and the London School of Hygiene & Tropical Medicine Ethics Committee (6432). Prior to the start of the study, sensitization meetings were held with teachers, parents, school management committees and traditional authorities. Following invitation of individual teachers to attend training by the District Education Office SHN Coordinator, an overview of the training, and risks and benefits of participation, were explained by the facilitation team and printed information sheets were provided to teachers to review. Written informed consent was obtained from all selected teachers before the beginning of both the pilot and the final programme training.

## Results

A total of 15 teachers participated in the pilot training and 92 teachers participated in the final training (Table 2), representing a wide range of ages and professional experience. In the final training, 67 % (*n* = 62) of participants were male and teachers were on average aged 30 years (head teachers 46 years). There were no significant differences in the characteristics of the sample of teachers evaluated as part of the pilot training, final training or at seven month follow-up (Table 2).

### Pilot training

**Fig. 1:**
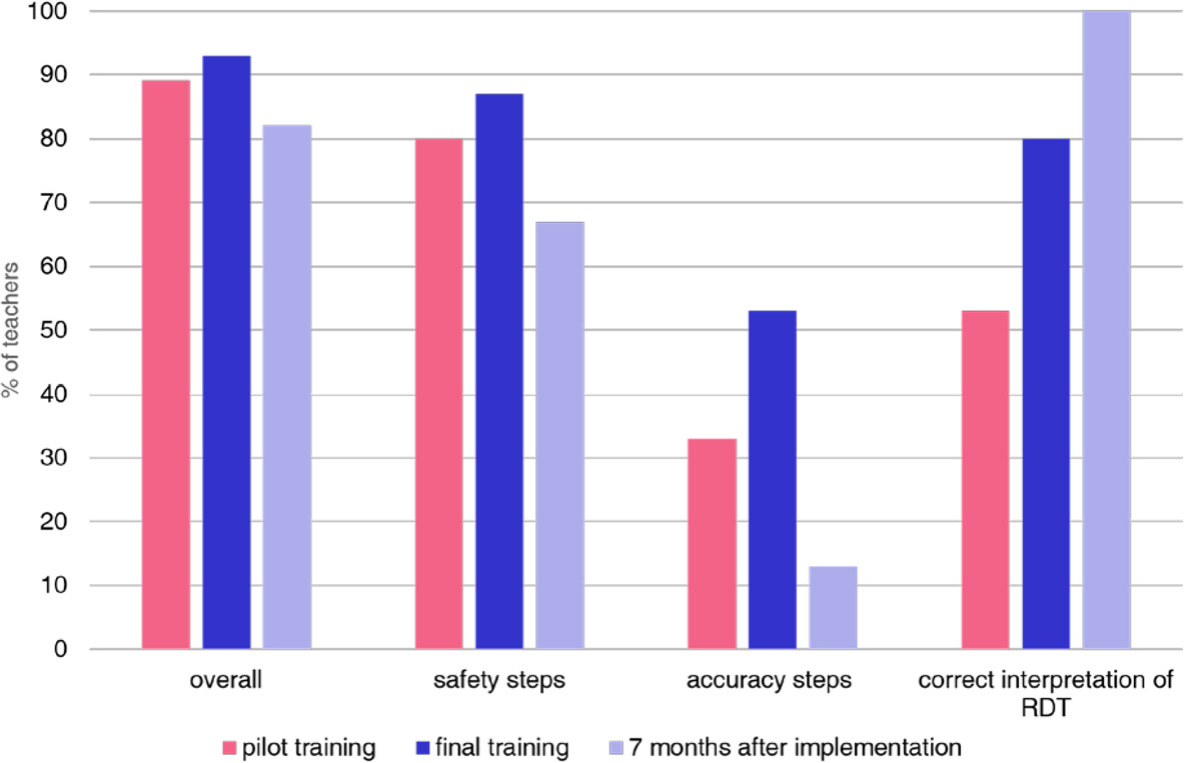
Percentage of teachers correctly completing (i) all RDT usage steps, (ii) all RDT steps defined as safe use (iii) all RDT steps defined as accurate use (iv) correct interpretation of RDT result

Table 3 details the proportion of teachers who completed each test step correctly during the pilot training and reports the most common errors. On average, teachers completed 89 % (standard deviation (sd) =1.54) of steps involved in the use of an RDT correctly (steps 1–21), with an average 2.33 (sd = 1.53) steps performed incorrectly and 0.93 (sd = 0.96) steps missed. The steps most frequently completed incorrectly were to check if the desiccant sachet was dry (step 5, 40 % incorrect) and to assemble all required equipment before beginning the RDT (step 2, 47 % incorrect). A total of 53 % of teachers identified all RDT results correctly (step 21), with the most common mistake misidentification of a faint positive or a negative result as invalid (Fig. 1).

While all teachers aged <37 years conducted all steps defined as safe use of RDTs, only 58 % of teachers aged ≥37 years did so (*p* = 0.07), with the most common error being placing the lancet down following use prior to disposal rather than immediate disposal of the lancet into the sharps container (step 12). There was no evidence to suggest that successful completion of all steps required to perform the RDT accurately differed significantly by age group (38 vs. 14 %, *p* = 0.57). However, correct reading of RDT results (step 21) was found to vary significantly between age groups (88 vs. 14 %, *p* = 0.01), with teachers aged <37 years performing better. The frequency of the other four most common errors did not vary significantly between age groups.

An average of 50 % of the five steps (step 21–26) associated with selection of appropriate treatment and correct completion of the treatment forms were completed correctly (Table 3). While 87 % of teachers completed the treatment register correctly, 60 % of teachers selected the appropriate artemether-lumefantrine (AL) and paracetamol doses for the child (frequently indicating in the treatment register the selection of a lower than required AL dosage) and only 13 % of teachers selected the correct parent information sheet for the AL dose required. Furthermore, less than half of the teachers stated the correct time for the second dose to be taken by the child (i.e., 8 h after the first dose).

Two weeks following training, the percentage of teachers correctly performing each step was the same or greater (Table 3). The most notable improvements observed were assembling the equipment prior to testing (step 1) (+40 %; *p* = 0.05) correctly interpreting the RDT result (step 21) (+35 %; *p* = 0.109). Checking the desiccant sachet (step 5, 66 %) and starting the timer immediately after adding buffer (step 18, 66 %) remained the two most commonly incorrectly completed steps at both assessments.

**Fig. 2:**
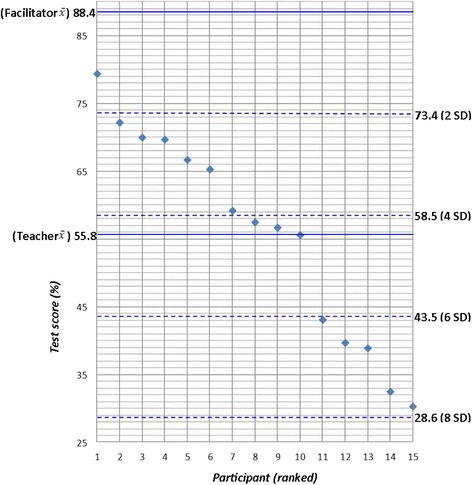
Script concordance test results ranked by teacher

When considering the ability of teachers to make appropriate clinical decisions (e.g., following a certain RDT result, the likelihood of a suggested diagnosis or suitability of a proposed treatment) compared to that of a hypothesised expert group (here represented by the facilitators) by SCT, there was strong evidence of a significant difference between the results of the two groups (*p* < 0.01). The mean SCT score for the expert panel was 88.4/100 (sd = 7.5) and 55.8/100 (sd = 14.9) for the teachers. In total, 47 % (*n* = 7) of participants were within four standard deviations of the expert panel mean score (Fig. 2).

### Perceptions of the pilot training by participants and facilitators

Teachers commonly praised the sessions where they were required to practice using an RDT as by far the most enjoyable or interesting component of the training programme. However, a lack of sufficient practical sessions was one of the most commonly described deficiencies of the pilot training by both facilitators and teachers, and perceived as key to improving both competence and confidence in the use of an RDT. All facilitators suggested that these practical sessions could have greater value if held in the programmatic setting.

*Others will also be very confident if they would change the time to have more practice. So if they are going to have more practice, all of them will be very confident.* Teacher 8, FGD 2.

*For them, practicing amongst themselves because they are all adults, it was somehow easier. They need to experience it with children, so they can learn how they can handle some children trying to run away and whatever, because this will be the real situation at their schools.* Facilitator 1.

The pilot training programme was considered to have been clear, relevant, and suitable to the background of participants. All facilitators commented on the fact that unlike previous RDT trainings that they had been involved in, this was the first where ‘non-medical’ professionals, with a different level of pre-existing knowledge and experiences, were being trained. All facilitators described training teachers as relatively more difficult to do as a result of a lack of both experience and confidence in carrying out “*medical procedures*”*:*

*It was a crucial component to bring them to the medical background part and make them understand what we really want to do…it was very easy to do the other training as compared to this teacher training.* Facilitator 2.

One facilitator, while still appreciating that this lack of familiarity made some aspects of training more difficult, suggested that it could actually improve the overall quality of the training programme. He described how the engagement of healthcare workers had been worse in previous trainings compared to that of the teachers, due to their greater familiarity with some of the concepts.

*This time when we were rolling out the rapid diagnostic tests [in health surveillance assistants] it was like a repeat, so people had some knowledge, the concentration was a little bit less because they had some knowledge.* Facilitator 3.

The majority of teachers (80 %) reported themselves as feeling ‘very confident’ in either the use of an RDT or treating for malaria in an anonymous post-training evaluation questionnaire. A smaller proportion (53 %) reported the same level of confidence in recognising ‘danger signs’ of malaria. Facilitators generally considered the training to have been successful in equipping participants with the necessary skills to manage minor illnesses and injuries competently. While acknowledging that it had not been the aim of the training programme to go beyond the referral of complicated illnesses, some reservations remained over the competency of teachers in this regard, echoing concerns implied by teachers’ lower reported level of confidence.

*It should be adequate, to manage minor illnesses at school. Because we are emphasizing the minor element, so it should be enough.* Facilitator 3.

### Final training

Following completion of the final training, teachers completed an average of 93 % (19.5/21) of RDT steps correctly (Table 4), a 4 % increase from the pilot training. With the exception of step 4 (checking the expiry date of the RDT) each step was correctly carried out by ≥80 % of teachers. When considered by steps necessary for safe or accurate use, 87 % of teachers completed the RDT safely and 53 % completed the RDT accurately (Fig. 1). With the exception of step 4, no individual safety or accuracy specific step was completed incorrectly by more than two teachers. A total of 80 % of teachers interpreted all RDT results correctly, a large but non-significant improvement from the pilot training (*p* = 0.25).

**Table 4:** Analysis of RDT use and learner treatment scenario steps (i) following completion of final training and (ii) 7 months after final training, by individual step

			(% completing step correctly) (n = 15)		
		Description of step	End of training^a^	7 months post training^b^	Most common error(s)
1		Work surface disinfected with alcohol	-	-	Not applicable due to change in training protocol.
2		Assembles all required equipment	87	100	
3	S	Put on new pair of gloves	93	93	
4		Check expiry date of RDT packet	67	80	Did not check.
5		Check desiccant sachet is dry	100	67	Did not check.
6		Write client name on cassette	80	93	
7		Place cassette on level surface	100	100	
8	S	Clean finger with alcohol swab	93	93	
9		Allow finger to dry before pricking	100	93	
10	S	Use sterile lancet to prick finger	93	93	Set down sterile lancet onto surface after removing cap
11		Puncture side of ball of the 3rd or 4th finger	100	73	Punctured vertically (i.e. perpendicular to the tip)
12	S	Dispose of lancet immediately after pricking finger	100	87	Used lancet, then set down onto work surface
13		Wipes away initial blood from finger	100	93	
14	A	Uses blood collection device correctly	100	100	
15	A	Correctly transfer blood to cassette using tool	100	100	
16	S	Dispose of blood collection tool immediately	100	87	Left blood collection device inserted into sample well.
17	A	Dispenses correct volume of buffer	87	60	Too much buffer added.
18	A	Start the timer immediately after adding buffer	87	60	Did not record time.
19		Dispose of non-sharps safely	93	100	
20a	A	States the correct time to read RDT (RDT test sheet)	100	-	
20b	A	Waits correct time before reading RDT result	-	27	Waited <5 minutes before reading result.
21	A	Reads test results correctly ^c^	80	100	
22	T	Completes correct sections of treatment register	87	93	
23	T	Selects appropriate AL dose based on weight	87	100	Did not record time.
24	T	Selects appropriate paracetamol dose	60	93	
25	T	Selects correct Parent Information sheet for AL dose	67	40	
26	T	States correct time for 2nd dose of AL to be taken	47	40	Did not complete information sheet, provided counselling verbally only

^a^observed at training venue, ^b^observed at school, *S* steps relating to safety of conducting test, *A* steps relating to accuracy of test, *T* steps relating to treatment of uncomplicated malaria ^c^The RDT conducted was also read for the evaluation of this step at the 7 month follow-up in addition to the printed sheet of photographed RDT results used for the evaluation of this step at the end of training

Provision of correct treatment (as assessed by case scenario) was observed to have improved when compared to the pilot training, most significantly in selection of the correct parent information sheet (step 25, 54 % increase, *p* = 0.007) but to a lesser extent also in selecting the correct dose of AL (step 23, 27 % increase, *p* = 0.11).

Following 7 months of LTK programme implementation, an average of 82 % of RDT steps were completed correctly (Table 4). The only step that significantly declined in correct completion was the proportion of teachers who checked whether the desiccant sachet inside the cassette packet was dry (33 % decrease, *p* = 0.04). When compared to the end of training, a higher proportion of teachers were observed checking the expiry date of the RDT (step 4, 13 % increase) and writing the child’s name on the cassette (step 6, 13 %). Declines in completion of individual steps were observed in the proportion of teachers correctly (i.e., to the side of the finger tip) puncturing the finger (step 11, 27 % decrease), dispensing correct volume of buffer (step 17, 27 % decrease) and starting the timer immediately after adding buffer (step 18, 27 % decrease). Only seven individual mistakes were recorded within a total of 75 safety steps conducted by the fifteen teachers at the seven month follow-up, and only two teachers failed on more than one safety component step (Table 5). Despite all participants correctly stating the correct length of time to wait before reading the result of an RDT and reading the RDT results correctly, only 27 % of teachers were observed waiting the correct length of time before reading the RDT result (Step 20b).

**Table 5:** Percentage of teachers correctly completing individual steps required to carry out an RDT (i) accurately or (ii) safely following final training and after 7 months after training

	Percentage of teachers completing number of steps correctly for accurate or safe RDT use (*n* = 15)			
	3 steps	4 steps	5 steps	6 steps
Accuracy at end of training	100	100	100	53
Accuracy 7 months post training	100	80	40	13^a^
Safety at end of training	100	93	87	
Safety 7 months post training	100	87	67	

^a^Only 2 teachers were observed correctly completing step 20 (waiting 20 min before reading the RDT result)

Notably improving relative to both the pilot and final trainings, all teachers after 7 months selected the correct dose of AL treatment, and all except one teacher provided the correct dosage of paracetamol (Table 4). While all teachers were observed providing instructions verbally on taking AL, 60 % of teachers did not provide the parent information sheet with correctly completed instructions (step 25 & 26). A significant increase in the percentage of teachers selecting the correct dosage of paracetamol by weight was observed (+33 %, *p* = 0.014) with no significant decline in any other treatment step.

## Discussion

This study reports the first known example of teachers being trained in the use of RDTs and identifies several important issues for the implementation of a programme involving the use of teachers to provide diagnosis and treatment of malaria in school children. While teachers were able to correctly carry out the majority of steps required to complete an RDT following a seven day training, and maintain this over the following seven months, there are specific areas – particularly the final steps of adding buffer solution, waiting the required length of time before reading, and some aspects of treatment administration – that have implications for monitoring of RDT use by teachers and future training involving similar participants.

While challenging to compare directly between studies owing to the variability of different RDT products and evaluation checklists used, the results of this study compare favourably with the findings of previous evaluations of RDT use following training, suggesting a similar level of competency in RDT use demonstrated by teachers compared to other RDT users when compared by the proportion of RDT steps completed correctly. An assessment of rural CHWs in Laos described an average of 78 % of steps involved in RDT use performed correctly with the use of a job aid plus one hour orientation [33], with two of the most common errors similarly relating to incorrect time waited before reading of the RDT result and a failure to check the condition of the desiccant sachet. Similar studies describe correct completion of all steps in the use of an RDT by 80 and 54 % of CHWs in Uganda respectively following a one-day training [10, 42]. The former of these studies again reported incorrect time waited before reading RDT results and both reference a similar common mistake relating to incorrect volume of buffer being added. While a study of CHWs has reported 90 % of steps were correctly completed using a job aid following a three-hour training [12], correct completion of steps required for ‘safe’ and ‘accurate’ use of the RDT was observed in 92 and 95 % of participants. In contrast, correctly completion of >95 % RDT steps has been observed among CHWs in the Democratic Republic of Congo [43] and Uganda [14]. Encouragingly, errors related to incorrect collection and transfer of the blood sample, frequently reported in several of these previous evaluations were not observed in the present study.

The performance of teachers in this study over the seven months following training declined significantly in two areas – failure to check the status of desiccant sachet, and waiting a correct length of time before reading the RDT result. A six-month evaluation of Sudanese nurses and medical assistants similarly identified inappropriate length of time waited before reading the test, but also inadequate blood volume and improper positioning of the device to be the most frequently observed errors when using an RDT [34]. An assessment of routine use of RDTs by 25 experienced South African nurses and nursing assistants, while noting that 76 % had only received ‘in-house’ training from their colleagues, specifically highlighted serious deficiencies in infection control aspects of RDT use, including failing to always use a new pair of gloves and disposal of the lancet immediately after pricking the finger [44]. In contrast, an assessment by Counihan *et al*. of Zambian CHWs at three month intervals following training observed high performance (87.5 % of ‘critical’ steps performed correctly at three months) which was maintained over 12 months [15].

Areas of concern following the pilot training – notably, failure to read RDT results correctly and provide appropriate treatment – appear to have been resolved following modifications to the final training programme. However, new errors in the volume of buffer added to the RDT, timing of the RDT and early reading of the RDT result arose over the following 7 months. The two later steps likely reflect inevitable consequences of short (15–30 min) school breaktimes in which to carry out a relatively high number of RDTs, a notable difference to the routine of RDT use by CHWs that may be more spread out over the course of the day. Step 20 of the checklist evaluation used during the pilot and final training was modified for use at follow up, requiring those conducting an RDT to wait 20 min (rather than stating the time to be waited) before reading the test result, regardless of whether it was positive or negative. We note that this study held a relatively strict definition of this component step compared to previous evaluations of RDT administration, such as allowing reading of a positive RDT result as soon as the control strip has appeared [15]. By this definition, 87 % of teachers would be considered to have correctly completed step 20 at the seven-month follow up.

The small evaluation sample size, while not unique to this specific study, restricts the power required to establish the significance of differences in performance. Furthermore, assessment in all but the last evaluation was observed in an artificial setting that simulated testing of a ‘child’ using another participant that may have biased performances. While the results of the SCT used in this study demonstrate its feasibility and potential usefulness in such a setting, several necessary concessions made in its design may have compromised its reliability. The SCT contained fewer cases than is usually accepted as required to achieve sufficient reliability [45] but still met the recommended one-hour length [46]. The size of the expert panel - restricted by the choice of facilitators as experts - was also smaller than required to achieve reliable scores [47], and the use of standard deviation to interpret scores has been criticised for its ultimately arbitrary nature [41]. The duration of training, and hence resources required, was considerably greater than previously reported trainings of RDT use owing to its incorporation into a larger first aid training programme. However, if conducted separately the RDT-specific component of this training would total approximately 2 full training days.

Through presenting the design, piloting, implementation and evaluation of a comprehensive training programme for teachers, our results further demonstrate the feasibility of training lay persons to manage uncomplicated malaria at community level using RDTs and ACTs. While this cohort of individuals is unique in taking on this task as a separate and additional responsibility, rather than change or modification to their primary role (such as in the case of healthcare workers or drug-shop keepers), we are able to identify common challenges raised by previous investigations of RDT use in other groups. However, this additional role also raises a number of questions, including the best methods of ensuring sufficient competency in the use of RDTs and ACTs in the longer term, and the ability of teachers to balance this role alongside their teaching schedule. These issues are being further investigated through an ongoing evaluation of the impact, cost effectiveness and acceptability of the programme of school-based diagnosis and treatment of uncomplicated malaria. Although the evidence of teachers’ ability to successfully carry out this role does not constitute an argument for replacing the functions of the formal health system, it does represent a novel and realistic opportunity to expand access to prompt diagnosis and treatment for school children within the context of a constrained health system.

## Conclusion

School teachers with no experience of providing malaria case management can be trained to carry out malaria rapid diagnostic tests safely and accurately and provide appropriate treatment as part of a seven-day training in management of basic illnesses and injuries. This competency can be maintained for at least seven months following training when teachers are conducting regular consultations of children in a school setting. Further investigation will determine whether this competency can be retained long-term, and the most effective combination of supervision, re-training and support to achieve this.
